# Prediction of strawberry yield based on receptacle detection and Bayesian inference

**DOI:** 10.1016/j.heliyon.2023.e14546

**Published:** 2023-03-13

**Authors:** Sunghyun Yoon, Jung Su Jo, Steven B. Kim, Ha Seon Sim, Sung Kyeom Kim, Dong Sub Kim

**Affiliations:** a Department of Artificial Intelligence, Kongju National University, Cheonan 31080, South Korea; b Department of Horticultural Science, College of Agricultural & Life Science, Kyungpook National University, Daegu 41566, South Korea; c Institute of Agricultural Science and Technology, Kyungpook National University, Daegu 41566, South Korea; d Department of Mathematics and Statistics, California State University, Monterey Bay, Seaside 93955, CA, USA; e Department of Horticulture, Kongju National University, Yesan 32439, South Korea

**Keywords:** Bayesian statistical analysis, Faster R–CNN, Object detection, Receptacle, Two seasons

## Abstract

The receptacle of strawberry is a more direct part than the flower for predicting yield as they eventually become fruits. Thus, we tried to predict the yield by combining an AI technique for receptacle detection in images and statistical analysis on the relationship between the number of receptacles detected and the strawberry yield over a period of time. Five major cultivars were cultivated to consider the cultivar characteristics and environmental factors for two years were collected to consider the climate difference. Faster R–CNN based object detector was used to estimate the number of receptacles per strawberry plant in given two-dimensional images, which achieved a mAP of 0.6587 for our dataset. However, not all receptacles appear on the two-dimensional images, and Bayesian analysis was used to model the uncertainty associated with the number of receptacles missed by the AI. After estimating the probability of fruiting per receptacle, prediction models for the total strawberry yield at the end of harvest season were evaluated. Even though the detection accuracy was not perfect, the results indicated that counting the receptacles by object detection and estimating the probability of fruiting per receptacle by Bayesian modeling are more useful for predicting the total yield per plant than knowing its cumulative yield during the first month.

## 1 Introduction

Growth models and prediction models have been developed for various crops. Most of the early growth models were developed for food crops, and research on the development of growth models for horticultural crops has been actively underway since the 2000s. In the Netherlands, growth models of major crops such as ORYZA, LINTUL, and SUCROS have been developed [41]. Reference [10] developed a model to predict the area of the entire leaf using the length of the middle leaf and the width of the left leaf of strawberries. Zadravec et al. [45] predicted the fruit diameter of apples. In addition to predicting plant growth, predicting the yield has gained attention as it is the most direct outcome of interest for farmers. References [12,13] used a regression method to predict strawberry yield using predictors such as weather data, fungicides and year of cultivation. Reference [29] combined simulations and machine learning algorithms to predict blueberry yield.

Strawberry is a high value-added crop with a worldwide production of 23 tons/ha [14]. Therefore, various growth and yield models have been studied. Logistic, Gompertz, and von Bertalanffy models were used to evaluate growth models and strawberry fruit productions [11], and environmental and growth data were used to predict strawberry growth [3,37]. Some studies have found that weather conditions are more important factors of strawberry yield than flowering and harvest periods [12,13]. In Norway, using historical yield data, a strong correlation was found between strawberry yield and fungicide [12], and the correlation between strawberry yield and temperature varied with the seasons [39]. Reference [25] developed a yield prediction equation using flower number and temperature data. In order to predict strawberry yield, it is necessary to understand the relationship between various parameters. However, most studies have relied on environmental and growth data, and models using strawberry images are lacking [25,37].

As imaging devices such as digital cameras have become cheaper and easier to install due to the improvement of technology, they are widely used in agricultural research. Studies using imaging devices are being conducted on various horticultural crops, and there are many studies on strawberries. References [23,46] have classified the maturation stages of strawberry flowers and fruits using multispectral images taken with a digital camera. Reference [44] acquired specific wavelength images of strawberries using a smartphone camera, and developed a non-destructive, accurate and convenient method for measuring strawberry maturation stages through a multivariate nonlinear model. Another study demonstrated the feasibility of strawberry yield prediction using image-derived variables [1].

In addition, recent studies have utilized artificial intelligence (AI), a technology that artificially implements human learning and perception abilities. Reference [27] developed a model for predicting strawberry yield by using vegetation index, soil characteristics, and plant parameters in an artificial neural network. Reference [30] developed an integrated system for monitoring strawberry hydroponic environment data and determining the harvest time using the IoT-Edge-AI-Cloud concept.

Image techniques have been used to detect targets and collect digital information from various horticultural crops [8,18], but they involve high costs. In this study, a common digital camera and object detection technique which are easier to use and more economic than the other imaging and AI techniques were used. Object detection is a technique for detecting object instances of a certain class (e.g., humans, cars, or buildings) in digital images and videos [9]. The technique has been used for detecting leaves, flowers, and fruits in horticultural areas. For example, in strawberry, an object detection technique which processes images through many layers using a R–CNN (region-based convolutional neural network) was developed to visually display the instances of flowers [21]. Reference [20] detected strawberry flowers in the outdoor field using Deep Neural Network, and the accuracy was 86.1%. A study was conducted to count the number of flowers through a region-based convolutional neural network for RGB images acquired through an unmanned aerial vehicle, and the accuracy was 84.1% [5].

Strawberry flower is a powerful factor for predicting the fruit yield because the flowers will become the fruit [21]. However, detecting flowers have several risks: (1) Strawberry petals are five which can be reduced by aging and disease, (2) the strawberry flower color is white, but it can be confused with the surrounding white objects, and (3) strawberry flowers can overlap with other flowers, which causes the uncertainty of the result of flower detection. Moreover, the receptacle is the more direct factor than the flower, although it could be more difficult to detect as it is smaller than the flower. Strictly speaking, the part that becomes the fruit is the receptacle, in the center of the flower, not the flower itself. To the best of our knowledge, however, there has been no study that detects the receptacle in a strawberry flower for the yield prediction so far.

To mitigate the risks and to predict fruit yield using a more direct factor, detecting the receptacle in a strawberry flower was considered. Strawberries have a yellow receptacle in the center of the flower to become the fruit unlike other plants, and the yellow color of the receptacle is less confusing with the surroundings when compared to the white color of strawberry flower. In this study, the number of receptacles were counted by combining the receptacle images of five strawberry varieties acquired during two cultivation periods with AI technology. One of the challenges is that not all receptacles are detected by the two-dimensional images, so it sometimes underestimates the actual number of receptacles. This uncertainty is estimated by Bayesian modeling, and several yield prediction models were compared.

Therefore, the objective of this study was to predict strawberry yield using R–CNN for receptacle detection and Bayesian modeling for uncertainty estimation. Furthermore, yield prediction models considering several factors may be proposed.

## 2 Material and methods

### 2.1 Strawberry cultivation and data collection

**Fig. 1:**
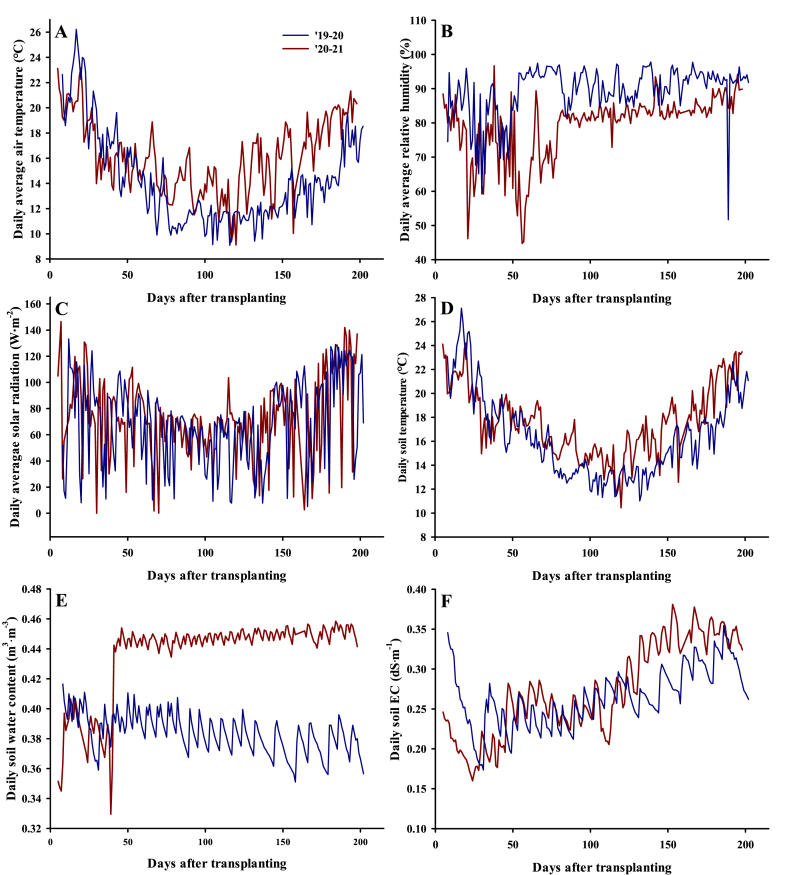
Environmental data collected from greenhouses during strawberry cultivation in the 2019–2020 and 2020–2021 trials (Blue line: 2019–2020, Red line: 2020–2021). A: Daily average air temperature; B: Daily average relative humidity; C: Daily average solar radiation; D: Daily soil temperature; E: Daily soil water content; F: Daily soil electrical conductivity (EC). (For interpretation of the references to color in this figure legend, the reader is referred to the Web version of this article.)

Five cultivars of strawberry were cultivated for this experiment including ‘Keumsil’, ‘Maehyang’, ‘Seollhyang’, ‘Arihyang’, and ‘Jukhyang’. ‘Seolhyang’ (84.5%), ‘Keumsil’ (4.1%), ‘Jukhyang’ (2.8%), and ‘Maehyang’ (2.5%) are cultivars that account for most of the distribution rate of domestic strawberries, and ‘Arihyang’ was recognized for its quality as an export cultivar. Strawberry seedlings were transplanted in two rows in a tunnel-type greenhouse located in the Kyungpook National University (35°53′N 128°36′E) at 18 cm intervals. The experiment was conducted using soil-fertigation cultivation and repeated twice over two years. Strawberry plants were cultivated using Japanese enshi nutrient solution in different concentrations between EC 1.0 and 1.5 dS m^−1^. The composition of the nutrient solution was 16, 1.34, 4, 8, 8, and 4 me L^−1^ of NO_3_–N, NH_4_–N, P, K, Ca, and Mg, respectively. The environmental data during the two trials are presented in Fig. 1.

**Fig. 2:**
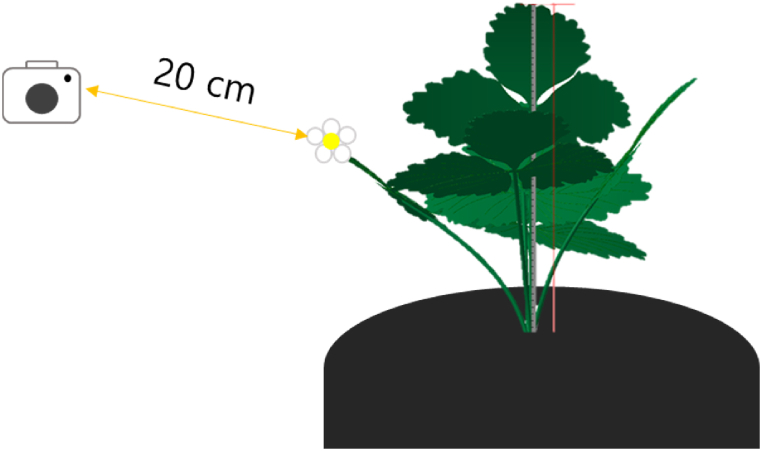
The camera location for photo data collection.

In the 2019–2020 trial, seedlings of ‘Keumsil’, ‘Maehyang’, and ‘Seollhyang’ were obtained from Kyungnam Agriculture Research Station (Jinju, Korea); ‘Arihyang’ seedlings were obtained from the National Institute of Horticulture and Herbal Science (Wanju, Korea); and ‘Jukhyang’ was purchased from Damyang-gun Agricultural Technology Center (Damyang, Korea). The first experiment was conducted from September 10, 2019 to April 2, 2020, and it was a randomized complete block design with five replications (22 strawberry transplants for each replication). ‘Keumsil’, ‘Maehyang’, and ‘Seollhyang’ were cultivated 205 days after transplanting (DAT); ‘Arihyang’ was cultivated 196 DAT; ‘Jukhyang’ was cultivated 189 DAT. ‘Seolhynag’, ‘Maehyang’, ‘Keumsil’, and ‘Arihyang’ had a difference in transplanting dates of about a week due to the difference in seedling supply timing, and ‘Jukhyang’ had different transplanting dates because it was a semi-forcing type. Flower images were taken using a Canon EOS 100D DSLR (Tokyo, Japan) once a week starting from October 16, 2019, the time of appearance of flower stalk. The flower images were taken in the direction and distance where the flower can be seen as best as possible (Fig. 2).

In the 2020–2021 trial, seedlings of Keumsil’, ‘Maehyang’,‘Seollhyang’, and ‘Arihyang’ were obtained from the same places as the first experiment, respectively; and ‘Jukhyang’ was purchased from a different seedling company in Damyang-gun (Damyang, Korea). The second experiment was conducted from September 14, 2020 to March 29, 2021, and it was also a randomized complete block design with five replications (20 strawberry transplants for each replication). ‘Keumsil’ was cultivated 196 DAT; ‘Maehyang’,‘Seollhyang’, and ‘Arihyang’ were cultivated 189 DAT; ‘Jukhyang’ was cultivated 172 DAT. ‘Seolhyang’, ‘Maehyang’, ‘Geumsil’, and ‘Arihyang’ had slightly different transplant days due to differences in seedling supply time, ‘Jukhyang’ was transplanted in early October because it is a semi-forcing type. Flower images were taken three times a week from November 16, 2020 in the same way as in the first experiment. Strawberries cultivated in the 2019–2020 season harvested ripe fruit 2–3 times a week from November 26, and strawberries cultivated in the 2020–2021 season harvested ripe fruit every day from December 10th. The number and weight of the harvested strawberries were measured for each cultivar.

### 2.2 Receptacle detection

The 1626 color images of (i.e.) pixels were used for our experiments, introduced above. The subsets were split into training and evaluation sets. The training set consists of 974 images collected during the period from December 18, 2020 to February 15, 2021. The evaluation set consists of 652 images collected during the period from November 6, 2019 to March 19, 2020. One of the authors manually labeled the receptacle(s) in each image of the training set with bounding boxes. The training set was used to train and validate the detection network, and model the relation between the number of receptacles and fruits. The evaluation set was used to estimate the probability of fruit production per receptacle, based on the inferred number of receptacles by the trained network.

Because all the images are RGB color images, they correspond to a three-dimensional array form with the shape of (C×H×W), where C is the number of channels (C=3 for RGB image), H and W are the height and width of each image, respectively (H=3456 and W=5184 in our experiments). An RGB image has pixel values ranging from 0 to 255. All the images to 400 × 600 were resized and then normalized all the pixel values by dividing by 255 to range from 0 to 1.

The state-of-the-art object detection methods can be categorized into two approaches: one-stage and two-stage approaches [40]. The one-stage methods, such as YOLO [35], are more concerned with inference speed than detection accuracy. Whereas the two-stage methods, such as Faster R–CNN [36], are more concerned with detection accuracy than inference speed. Because accuracy is generally more important than the speed in long-term horticultural applications, Faster R–CNN was used for the receptacle detection.

Faster R–CNN is an extension of Fast R–CNN [15] by means of a region proposal network (RPN). An RPN enables efficient and accurate region proposal generation with nearly cost-free computation, by sharing convolutional features with the detection network (corresponding to a backbone). The RPN module generates object region proposals by sliding a spatial window over the output feature map from the backbone. Both the backbone and RPN are trained in an end-to-end manner (i.e., not separately trained). A ResNet50 [16] was used as the backbone of the Faster R–CNN. A detailed structure of ResNet50 is described in [16]. To alleviate the data scarcity problem, the ResNet50 pre-trained on the COCO dataset [22] was used, a popular large-scale object detection dataset. The pre-trained network is available on Torchvision package [26]. The whole network was then fine-tuned for 10 epochs using the training set of the strawberry images. The SGD optimizer was used for fine-tuning with a batch size of 2, learning rate of 0.005, momentum of 0.9, and weight decay of 0.0005. The learning rate was decayed by 0.1 for every 3 epochs. PyTorch [32] was used to implement the receptacle detection network.

**Table 1:** Estimated regression parameters and inferential statistics.

M1	Estimate	SE	T	p-value	AIC	Adj-R^2^	MSE
Intercept	14.667	1.629	9.005	<0.0001	495.9	0.235	42.6
J	2.933	2.303	1.273	0.2071			
K	7.933	2.303	3.444	0.0010			
M	3.933	2.303	1.708	0.0921			
S	10.667	2.303	4.631	<0.0001			
M2	Estimate	SE	T	p-value	AIC	Adj-R^2^	MSE
Intercept	10.683	1.699	6.289	<0.0001	478.9	0.398	34.1
J	6.917	2.230	3.101	0.0028			
K	6.115	2.084	2.934	0.0045			
M	5.838	2.088	2.796	0.0067			
S	9.021	2.077	4.344	<0.0001			
*frt*	36.369	8.152	4.461	<0.0001			
M3	Estimate	SE	T	p-value	AIC	Adj-R^2^	MSE
Intercept	15.692	2.591	6.055	<0.0001	465.3	0.504	28.1
J	5.128	1.906	2.690	0.0090			
K	3.129	2.436	1.285	0.2033			
M	3.929	2.113	1.860	0.0673			
S	7.483	2.130	3.513	0.0008			
*logit(prob)*	4.117	0.734	5.611	<0.0001			
*rec*	8.792	2.817	3.121	0.0026			
M4	Estimate	SE	T	p-value	AIC	Adj-R^2^	MSE
Intercept	13.320	2.771	4.807	<0.0001	462.5	0.528	27.0
J	5.154	1.861	2.770	0.0072			
K	3.289	2.378	1.383	0.1713			
M	3.948	2.062	1.915	0.0598			
S	7.020	2.090	3.358	0.0013			
*logit(prob)*	2.008	1.235	1.626	0.1086			
*rec*	14.864	3.995	3.721	0.0004			
*rec* × *logit(prob)*	4.724	2.255	2.095	0.0399			

Due to the limited amount of the bounding-box-labeled strawberry images, the detection accuracy of the network based on 10-Fold cross validation was validated. The mean Average Precision (mAP) [7] was used as the detection evaluation metric, which is one of the primary metrics on object detection. The mAP is computed by averaging multiple values of average precision (AP). The APs are calculated with different Intersection over Union (IoU) thresholds, where the IoU is the ratio of the overlap and union areas between the ground-truth and predicted bounding boxes for an object. It is considered to successfully detect if the IoU is above a pre-defined threshold. The IoU thresholds range from 0.5 to 0.95 at an interval of 0.05. The mAP ranges from 0 to 1 and a higher mAP stands for higher detection accuracy.

### 2.3 Statistical analysis

The goal of statistical analysis was to predict the total number of strawberry fruits at the end of the harvest season. To obtain a strawberry fruit, a plant must have a receptacle, but not all receptacles produce a strawberry fruit. In this regard, it is important to estimate both the number of receptacles and the probability of fruiting per receptacle.

Let n_obs_ be the total number of receptacles detected by the AI based on pictures taken from December 18, 2020 to February 15, 2021 (one picture per plant). Note that not all receptacles were captured by the pictures, so the true total number of receptacles would be greater than n_obs_. Let N = n_obs_ + n_mis_ be the true number of receptacles, where n_mis_ is the total number of receptacles undetected by the AI. To account for the uncertainty of N, we modeled n_obs_ by a binomial distribution with parameters N and θ, where θ is the unknown proportion of receptacles (per plant) detected by AI. Note that θ is estimable when we have an observed sample of N. We randomly selected 4 plants per cultivar and recorded both n_obs_ and n_mis_ by counting the number of receptacles that a two-dimensional picture captured and did not capture for each plant. This data, combined with the uniform (noninformative) prior on θ, was used to model the posterior distribution of θ (the beta distribution with the shape parameters α = 48 and β = 22), and we obtained the posterior predictive distribution of N per plant.

We let Y be the total number of fruits per plant, and let π be the probability of fruiting per receptacle. Given N, we used a quasi-binomial distribution (to account for over-dispersion in the count data) and obtained the posterior distribution of π per each cultivar.

Farmers' primary interest is to foresee the total productivity at the end of harvest season, and it depends on the total number of plants in a farm. In this study, we focused on the prediction of the number of fruits per plant using the information available early in the harvest season. We used the first four weeks of data (from December 18, 2020 to January 15, 2021) to model the number of fruits per plant.

For the purpose of concise presentations, we define the following notations. We let μ be the expected number of fruits per plant as of March 26; *frt* and *rec* be the rate of change in the average number of fruit and of receptacles per plant, respectively, observed from December 18 to January 15; and *prob* be the probability of fruiting per receptacle with the logistic transformation. We then compared four multiple regression models: (M1) μ explained by the cultivar only; (M2) μ explained by the cultivar and *frt*; (M3) μ explained by the cultivar, *rec* and *prob*; and (M4) μ explained by the cultivar, *rec*, *prob*, and *rec* × *prob* (i.e., the interaction between *rec* and *prob*).

The four models (M1 to M4) were evaluated by the Akaike Information Criterion (AIC) and the adjusted-R^2^, and their predictive performances were evaluated by the mean square error (MSE) estimated from the leave-one-out-cross-validation (LOOCV).

## 3 Results

**Fig. 3:**
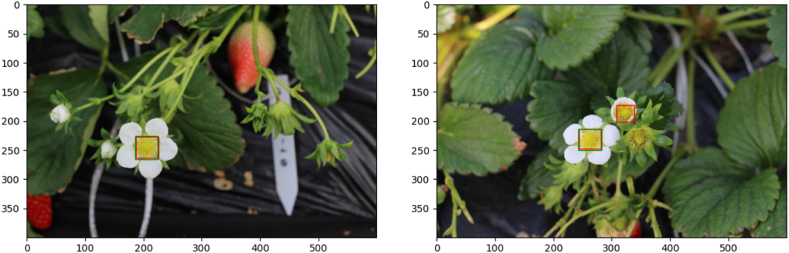
Detection of the receptacle in photographic data. Green rectangle: Receptacle detected by a human, Red rectangle: Receptacle detected by AI from learning data. The picture on the left displayed good agreement between detection by humans and AI, but the picture on the right indicated better detection by AI. (For interpretation of the references to color in this figure legend, the reader is referred to the Web version of this article.)

Fig. 3 illustrates two common cases on the receptacle detection. Ground-truth and predicted bounding boxes are represented in green and red, respectively. The receptacle detection is not always perfect. For instance, there was a case where a bee on a receptacle is detected as a receptacle, and there were some cases where the model predicts other objectives (e.g., a leaf) as a receptacle. The left of Fig. 1, Fig. 3 stands for the case where the network correctly detected the receptacle(s) in an image. Most results correspond to this case, which means the network was well-trained to detect the receptacle. In contrast, the right of Fig. 1, Fig. 3 stands for the case where the network correctly detected not only the labeled but unlabeled receptacles. In the training set, 156 out of 974 training images correspond to this case. Therefore, the somewhat low detection accuracy of mAP 0.6587 would be mainly due to the problem of human mislabeling, and the actual detection accuracy of the network would be higher than that reflected by the mAP of 0.6587. The estimated correlation between AI count and human count was 0.856 (p < 0.0001). When individual images were analyzed, the AI captured 0.19 more receptacles than human, on average.

**Fig. 4:**
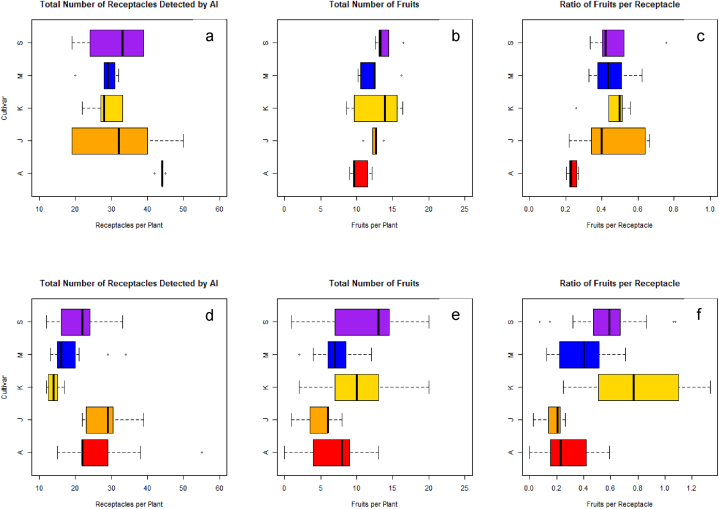
Total number of receptacles detected by AI (a and d), total number of fruits (b and e), and ratio of fruits per receptacle (c and f) of five strawberry cultivars grown in the 2019–2020 (a, b, and c) and 2020–2021 (d, e, and f) trial. A: ‘Arihyang’, J: ‘Jukhyang’, K: ‘Keumsil’, M: ‘Maehyang’, and S: ‘Seolhyang’.

For the two seasons, five strawberry cultivars including ‘Arihyang’, ‘Jukhyang’, ‘Keumsi’, ‘Maehyang’, and ‘Seolhyang’ were studied. In the 2019–2020 season, the number of receptacles per plant of ‘Arihyang’ were statistically significantly more than those of the other cultivars (p = 0.0002) while fruits per plant tended to be less (p = 0.0603) (Fig. 4a–c). The trends led to the result with the lowest fruits per receptacle (p = 0.0036). ‘Jukhyang’, ‘Keumsi’, ‘Maehyang’, and ‘Seolhyang’ showed similar averages for the number of receptacles per plant, fruits per plant, and fruits per receptacle.

In the 2020–2021 season, the average numbers of receptacles per plant of ‘Arihyang’ and ‘Jukhyang’ were statistically significantly higher than those of the other cultivars (p < 0.0001) while fruits per receptacle were statistically significantly less (p = 0.0003). The results, as in the 2019–2020, displayed that the low ratio of fruits per receptacle is a characteristic of ‘Arihyang’. The receptacles per plant, fruits per plant, and fruits per receptacle of ‘Keumsi’, ‘Maehyang’, and ‘Seolhyang’ were similar as in the 2019–2020 (Fig. 4d–f).

**Fig. 5:**
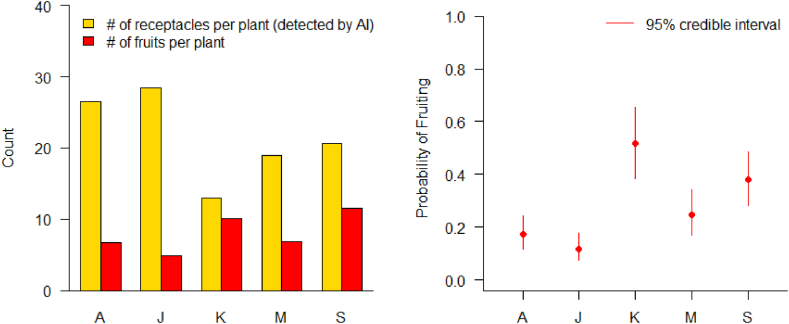
The average number of receptacles and of fruits per plant (left) and the estimated probability of fruiting per receptacle (right) by cultivar.

The AI tended to under-estimate the number of receptacles because not all receptacles can be captured in two-dimensional images. Using the Bayesian analysis described in the statistical analysis section, we accounted for the uncertainty associated with the number of missed receptacles, and Fig. 5. Presents the estimated probability of fruit production per receptacle for each cultivar. The results seem clear that ‘Arihyang’ and ‘Jukhyang’ produce more receptacles but are not efficient (i.e., low probabilities of fruiting per receptacle). On the other hand, ‘Keumsil’ produces less receptacles, but it is very efficient in terms of the probability of fruit production.

Four regression models with the following sets of predictors were compared. Model 1 (M1) considered the cultivar only as a predictor, M2 used the cultivar and the fruit yields during the first month of harvest, M3 used the cultivar, the estimated number of receptacles (rec), and the estimated probability of fruiting per receptacle with logistic transformation (prob), and M4 added a multiplicative term rep × prob in the model. The respective adjusted R-square was 0.235, 0.398, 0.504, and 0.528, and the respective mean square error was 42.6, 34.1, 28.1, and 27.0 estimated by the LOOCV. Among the four models compared, M4 was the best which indicates that the effect of having more receptacles depends on the probability of fruiting, which is a sensible result. In addition, the statistical results indicate that the number of receptacles and the probability of fruiting are more helpful information for yield prediction than knowing the first-month yield prediction (Table 1).

## 4 Discussion

The characteristics of the tested five cultivars have been investigated during 2019–2020 and 2020–2021 seasons [3,17]. In the two studies, ‘Arihyang’ displayed the greatest average fresh weight per fruit among the five cultivars. It may be due to a lower ratio of fruits per receptacle than the other cultivars except ‘Jukhyang’ which was transplanted later than the other cultivars. Reference [19] reported that the supply of carbon-based compounds available to fruit may be limited by competition from too many sinks during early fruit development. Since flowers or fruitlets can be the sink, the fruit of ‘Arihyang’, which had more flower that did not become fruit than the other cultivars, may have been larger than the fruit of the other cultivars. Therefore, identifying the characteristics of each cultivars may provide information for predicting strawberry fruit yield according to the cultivars.

Many researchers have reported that object detection can work much faster than humans. Among the object detection techniques, Ref. [42] displayed that the faster R–CNN test-time speed of object detection algorithms we used was 0.2 s, compared to 49 s for R–CNN and 2.3 s for fast R–CNN. Moreover, in our experiments, the detection model successfully detected some receptacles that should be detected but were not detected by humans, suggesting that AI can detect even objects that humans miss. Object detection technologies are advancing rapidly, the accuracy of the detection will be improved. Moreover, with the dramatic increase in the capability, sophistication, and miniaturization of imaging sensors, a lot of digital information will be collected in horticultural area [8,18,38]. But many studies have been conducted in a short period and there are few examples of long-term applications of the technologies in agriculture although most crops take several months to grow and harvest. For instance, Refs. [5,21] developed a strawberry flower detection system for fruit yield prediction using an unmanned aerial vehicle (UAV), digital or RGB camera, and object detection technique but the image data were collected once or every two weeks for four months in the studies. Furthermore, characteristics of various cultivars were not considered and actual fruit yield to predict total fruit yield has not been continuously monitored. On the contrary, the flower images and actual fruit yield during two years with five cultivars were collected in this study.

The limitation of this study is that the receptacle is likely to be obscured like the flower, and the object detection technique missed some receptacles due to two-dimensional photos for model training. We tried to address this caveat by the Bayesian modeling and quantified the uncertainty, and more training data could reduce the uncertainty in the posterior analysis of the Bayesian modeling. Future studies can address the caveat by a bigger training dataset and training multiple photos per plant (from multiple dimensions) to improve the object detection algorithm. Otherwise, if it is difficult to collect much more labeled photos, especially for training, both semi-supervised and self-supervised learnings would become breakthroughs against the data scarcity problem [6]. Although the data scarcity problem was alleviated by using the pre-trained detection model on a large-scale dataset of COCO, there is still a domain mismatch problem. Because the COCO dataset does not contain strawberry images, it is somewhat difficult to say that our object detector, pre-trained on the COCO dataset and then fine-tuned with a small number of strawberry images, is sufficiently optimized for receptacle detection. To this end, some methods based on semi-supervised [24] and/or self-supervised [31] learnings can be considered in future, which are beyond the scope of this paper. The detailed and accurate information about the receptacle is scientifically plausible because the receptacle is the only part that becomes the fruit and the health and maturity level of the flower can be identified and filtrated by the color or shape of the receptacle. In addition to the number of fruits and of receptacles observed for the first four weeks of the harvest season, growth variables (e.g., the number of leaves, leaf length and width, and crown size), environmental variables (e.g., day length and light quality), and detailed factors associated with the quality of receptacle (e.g., receptacle color and shape) may improve the yield prediction in future studies.

Many researchers have designed models and have used the models to actual production processes [2,4,5,28,33,34,43]. For example, when the growth of a horticultural crop is monitored, it may be judged by the designed models so that optimal management decisions might be possible to optimize the growth process. The recent studies [3,17] showed that the fruit yield depends on cultivars, and this current study further provided statistical evidence that the number of receptacles and the probability of producing fruit per receptacle are characteristics of cultivar as well. We used the object detection technique, and we anticipate that advanced objection detection techniques (e.g., accurate count of receptacles by using photos of multiple angles) can improve the predictive modeling with less manual efforts. As we continue this line of research, we expect more benefits from Bayesian modeling by incorporating more prior information.

## 5 Conclusion

A combination of an AI technique and statistical strategies was used for yield prediction in strawberry images with uncertainty. The combination achieved a mAP of 0.6587 and proposed models with higher R^2^ and lower MSE and AIC. The results indicated that collaboration with AI engineers and statisticians may make a great contribution to predicting strawberry yield. However, we recognized that complementary works such as photo data with mostly well-taken flowers, more photo data to improve efficiency of AI, and application of automatic photography technology should be needed on data collection to improve the accuracy of the prediction. Moreover, future studies with additional growth and environmental variables and receptacle color and shape may overcome the weakness of our data.

## Author contribution statement

Sunghyun Yoon: Conceived and designed the experiments; Performed the experiments; Analyzed and interpreted the data; Wrote the paper.

Jung Su Jo: Performed the experiments; Analyzed and interpreted the data; Contributed reagents, materials, analysis tools or data; Wrote the paper.

Steven B Kim: Conceived and designed the experiments; Analyzed and interpreted the data; Wrote the paper.

Ha Seon Sim: Performed the experiments; Contributed reagents, materials, analysis tools or data.

Sung Kyeom Kim & Dong Sub Kim: Conceived and designed the experiments; Contributed reagents, materials, analysis tools or data; Wrote the paper.

## Funding statement

Sung Kyeom Kim was supported by National Research Foundation of Korea [2019R1I1A3A01063693]; Dong Sub Kim was supported by Kongju National University [2022].

## Data availability statement

Data included in article/supplementary material/referenced in article.

## Additional information

No additional information is available for this paper.

## Declaration of interest's statement

The authors declare no conflict of interest.
